# Optical and electrical properties of the nanodisk-shaped SnS layers grown by sputtering

**DOI:** 10.1016/j.dib.2017.09.037

**Published:** 2017-09-21

**Authors:** Malkeshkumar Patel, Joondong Kim

**Affiliations:** Photoelectric and Energy Device Application Lab (PEDAL), Department of Electrical Engineering, Incheon National University, Incheon 406772, Republic of Korea

## Abstract

In this data article, we presented the structural, optical, and electrical data of the nanodisk-shaped SnS layers. A facile formation of orthorhombic SnS derived from SnS_2_ particles was discussed in our previous study (Patel et al., 2017) [1]. The data includes the standard XRD patterns supercell structure of the Orthorhombic SnS material, the photograph of prepared samples, thickness dependent absorbance spectra, and temperature dependent carrier concentration and its mobility estimated from the hall measurement of SnS samples.

**Specifications Table**

**Table:** 

Subject area	*Physics, Chemistry, Electrical Engineering*			
More specific subject area	*Solar Material*			
Type of data	*Photograph, Figures*			
How data was acquired	*Crystallography open data base and crystallographic tool – Diamond : Crystallographic Information File Code: 9008785.cif* (http://www.crystallography.net/cod/9008785.html)			
	*UV-visible diffused reflectance (UVDRS) spectrophotometer (Shimadzu, UV-2600)*			
	*Digital camera: Embedded cell phone camera (Motorola X)*			
	*Hall measurement unit: (Ecopia Hall Effect Measurement System, HSM-5300)*			
Data format	*Analyzed*			
Experimental factors	*Unit cell and standard XRD pattern:*			
	COD-9008785, Pseudo-Voigt profile function (*w*=0.6), 2theta step=0.01°, FWHM=0.4°, base width=10°, Lorentz and Polarization factors: enabled. Tool: Diamond.			
	Super cell: 5×5×5			
	*Photograph of Sample: Day light*			
	*UV-visible diffused reflectance (UVDRS) spectrophotometer:*			
	*Absorbance-*	*Baseline:*		*FTO/glass substrate,*
		*Scan range:*		*1440 nm to 270 nm*
		*Speed:*		*medium*
		*Scan step:*		*1 nm*
	*Hall measurements: Contact:*		*Ag paste*	
			*Current – 100 uA*	
			*Temperature – 300 K – 375 K*	
			*Thickness 100 nm*	
			*Magnetic Field: 0.552 T*	
			*Step: 5*	
			*Interval: 1*	
Experimental features	*Nanodisk-shaped SnS layered sample, thickness variation, Semitransparent SnS samples,* standard XRD pattern and super cell presentation			
Data source location	*Incheon National University, Incheon 22012, Korea*			
Data accessibility	*The data are with this article*			

**Value of the data**

• Rietveld refined XRD pattern of nanodisk-shaped SnS sample confirmed the orthorhombic crystalline structure of few-layered SnS material [1].
• Thickness-dependent optical properties of nanodisk-shaped SnS sample would be useful to explore the SnS as a photonic material.
• Temperature dependent carrier concentration and its mobility data provide the information for the electrical properties of nanodisk-shaped SnS material. These data would be valuable to explore the transport properties of this material.

## 1 Data

**Fig. 1:**
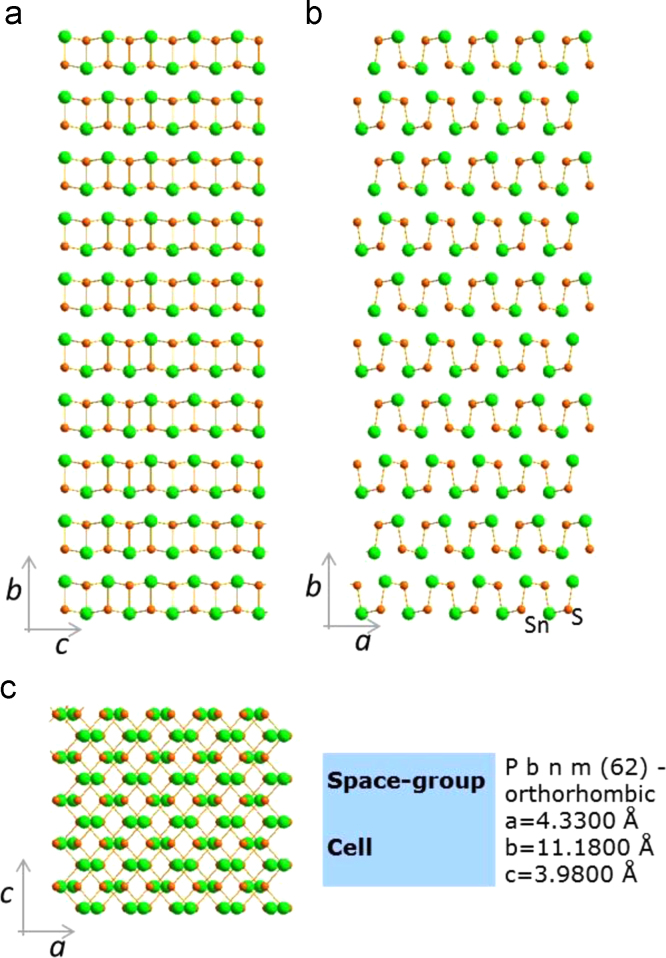
(a) Orthorhombic supercell of SnS material. Along lattice axis (a) *a*, (b) *b* and (c) *c.* This supercell consist of 5×5×5 unit cells. This structure was prepared using the COD-9008785.cif. This file can be available on the crystallographic open database.

**Fig. 2:**
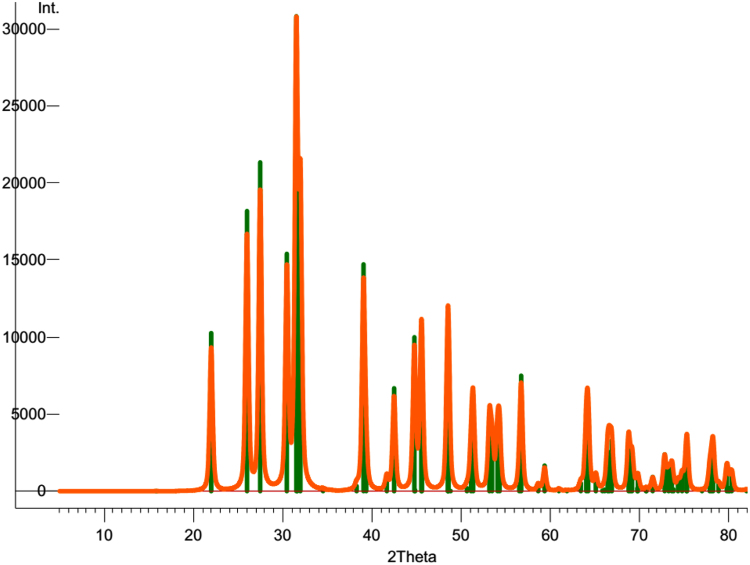
Standard XRD pattern of orthorhombic SnS material generated using the crystallographic information file COD-9008785 with Pseudo-Voigt profile function (*w*=0.6) with 2 theta step=0.01°, FWHM=0.4°, base width=10°, with enabled Lorentz and Polarization factors. Diamond tool was used to generate a standard XRD pattern.

**Fig. 3:**
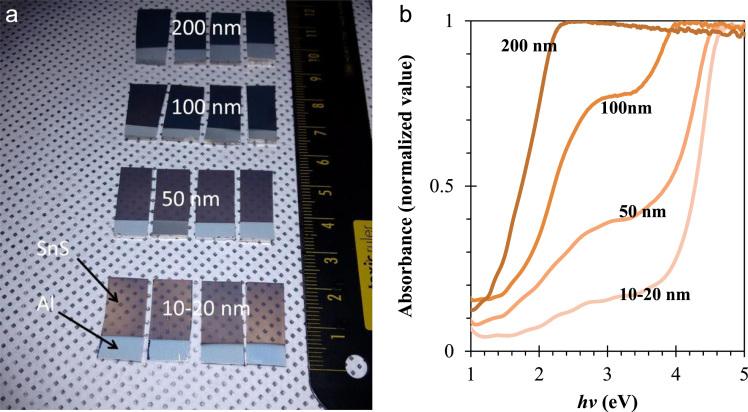
(a) Photograph of the prepared samples of SnS material on the FTO/glass substrate. (b) Absorbance characteristics of the SnS samples.

**Fig. 4:**
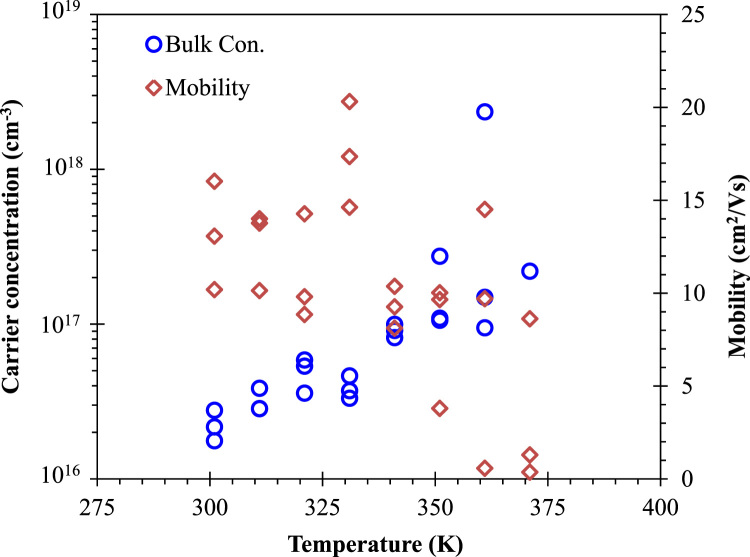
Hall measurement of 100 nm thick SnS sample on the Quartz wafer. Estimated carrier concentration (Acceptor, Bulk Con.) and hole mobility as a function of measured temperature are shown.

Fig. 1 shows the super cell presentation of the orthorhombic SnS material of pace group *Pbnm*(62) having the unit cell dimension as *a*=4.33, *b*=11.18 and *c*=3.98 Å. This structure was prepared using the crystallographic open data COD file 9008785. Standard XRD pattern of orthorhombic SnS material generated using this file with Pseudo-Voigt profile function with 2theta step=0.01°, FWHM=0.4°, base width=10°, with enabled Lorentz and Polarization factors is shown in Fig. 2. Semitransparent SnS samples of various thicknesses are shown in Fig. 3a. Absorbance data as a function of photon energy (*hv*) these samples are shown in Fig. 3b. SnS samples of various thicknesses were prepared by controlling the deposition time as detailed in the reference [1]. Temperature dependent Hall-measurement of 100 nm thick SnS sample prepared on the quartz wafer is shown in Fig. 4.

## 2 Experimental design, materials and methods

### 2.1 Preparing SnS samples

SnS films were grown on the quartz wafer and the FTO/glass. These substrates were cleaned in a series of chemical baths containing isopropyl alcohol, acetone, and distilled water using ultrasonication before SnS film growth. RF sputtering power of 50 W was applied to the SnS_2_ target (SnS_2_, 99.999%, iTASCO, TSNALT0027) to shower the SnS_2_ particles. These particles undergo the phase structural transition to form the SnS material [1]. This process was performed at 6 mTorr of working pressure under the flowing Ar (50 sccm) gas at the substrate temperature of 400 °C. The growth rate of SnS film was 5 nm per minute. Various thickness of SnS samples was obtained by controlling the deposition time.

Optical characteristics of SnS samples were measured using by UV-visible diffused reflectance (UVDRS) spectrophotometer (Shimadzu, UV-2600). Hall measurement was performed by using the Hall measurement system from Ecopia. Ag paste was applied to make Ohmic contact to 100 nm thick SnS sample. The temperature was varied from 300 K to 375 K with appropriate shield and device under test unit.
