# Supplementary material for: Optical and electrical properties of the nanodisk-shaped SnS layers grown by sputtering
Source: Data Brief. 2017 Sep 21;15:252–6. doi: 10.1016/j.dib.2017.09.037 (PMC5635201; doi:10.1016/j.dib.2017.09.037)
Supplement: Supplementary file 1 — Transparency document [file mmc1.docx]

***Conflicts of Interest Statement***

Re: DIB-D-17-00578R1

Title: *Optical and electrical properties of the nanodisk-shaped SnS layers grown by sputtering*

We declare that this manuscript is original, has not been reported before, and is not currently being considered elsewhere. We also confirm that there is no known conflict of interest regarding this manuscript and its publication. The manuscript has been approved by all named authors.

Sincerely yours,


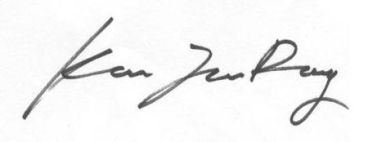


Joondong Kim

Joondong Kim, Ph.D./Professor

Department of Electrical Engineering,

Incheon National University

E-mail: joonkim@ incheon.ac.kr

Phone: +82-32-835-8770; fax: +82-32-835-0773
